# Use of Photoreactive Riboflavin and Blue Light Irradiation in Improving Dentin Bonding—Multifaceted Evaluation

**DOI:** 10.3390/jfb16010011

**Published:** 2025-01-03

**Authors:** Ping-Ju Chen, Jung-Pei Hsieh, Hsiao-Tzu Chang, Yuh-Ling Chen, Shu-Fen Chuang

**Affiliations:** 1 Department of Dentistry, Changhua Christian Hospital, Changhua 50006, Taiwan; 139370@cch.org.tw; 2 School of Dentistry and Institute of Oral Medicine, College of Medicine, National Cheng Kung University, Tainan 70101, Taiwan; z10207004@email.ncku.edu.tw (J.-P.H.); nakawsawaiy@gmail.com (H.-T.C.); yuhling@mail.ncku.edu.tw (Y.-L.C.); 3 Department of Stomatology, National Cheng Kung University Hospital, Tainan 70403, Taiwan

**Keywords:** riboflavin, dentin bonding, collagen crosslinking, enzymatic degradation, nanoindentation, microtensile bond strength

## Abstract

Recently, photoactivated riboflavin (RF) treatments have been approved to improve resin–dentin bonding by enhancing dentinal collagen crosslinking. This study aimed to evaluate whether RF activated by blue light (BL, 450 nm) strengthens the collagen matrix, increases resistance to enzymatic degradation, and improves adhesion as effectively as ultraviolet A (UVA, 375 nm) activation. Six groups were examined: control (no treatment); RF0.1UV2 (0.1% RF with 2 min of UVA irradiation); RF0.1BL1, RF0.1BL2, RF1BL1, and RF1BL2 (0.1% and 1% RF with 1 or 2 min of BL irradiation). The effects of RF/BL on collagen crosslinking were validated by gel electrophoresis. A nanoindentation test showed that both RF/UVA and RF/BL treatments enhanced the elastic modulus and nanohardness of demineralized dentin. A zymography assay using collagen extracted from demineralized dentin also revealed significant matrix metalloproteinase-2 inhibition across all RF treatments. Microtensile bond strength (µTBS) tests conducted both post-treatment and after 7-day enzymatic degradation showed that three RF0.1 groups (RF0.1UV2, RF0.1BL1, and RF0.1BL2) maintained high µTBS values after degradation, while RF0.1BL1 generated a significantly thicker hybrid layer compared to other groups. These findings suggest that RF/BL is as effective as RF/UVA in crosslinking dentinal collagen and resisting enzymatic degradation, with 0.1% RF proving superior to 1% RF in enhancing dentin bonding.

## 1. Introduction

Contemporary dental bonding systems rely on the formation of a hybrid layer (HL) on acid-etched dentin to achieve successful adhesion [1]. Dentin bonding can be considered a form of tissue engineering [2], as the microstructure of the HL is created by infiltrating resin monomers into the collagen matrix of the demineralized dentin and polymerizing them in situ. The resulting micromechanical interlocking not only provides retention for resin restorations but also sustains functional occlusal loading and resists biological degradation. Unlike classical tissue engineering, the HL is expected to form a tight and permanent connection between dentin and resin composites, rather than being replaced by natural dentin. To fulfill this purpose, the HL should be structurally stable.

Most dentin adhesives exhibit high immediate bond strengths to counteract polymerization shrinkage stress [3]. However, achieving successful long-term dentin bonding remains a challenge. For many etch-and-rinse adhesive systems, the primary issue lies in maintaining the optimal presence of water, which is essential for expanding the denuded collagen matrix and facilitating the penetration of adhesive monomers to form an intact HL [4]. Unsupported collagen collapses during air-drying, thereby impairing resin infiltration and leaving vacancies at the bottom of the HL, which can lead to nanoleakage [5,6]. The lack of resin protection and the presence of water render the unembedded collagen fibrils vulnerable to hydrolytic degradation, creating a weak joint under functional stress [7,8,9]. The durability of the HL is also compromised by biodegradation caused by matrix metalloproteinase (MMPs). Etching with acid or acidic monomers activates intrinsic MMPs within dentin, while collagen fibrils in the mineral-depleted spaces are particularly susceptible to enzymatic degradation [9,10,11].

To overcome these problems, biomodifications of the collagen matrix in the HL have been proposed as a strategy. A variety of crosslinking agents have been suggested to enhance the physicomechanical properties of collagen networks, improve the structural integrity of the resin–dentin interface, and resist enzymatic degradation [12]. Glutaraldehyde is a well-known collagen crosslinker, but its cytotoxicity is a concern [13]. Other natural or synthetic crosslinking agents, such as proanthocyanidins, genipin, carbodiimide, ethylenediaminetetraacetic acid (EDTA), benzalkonium chloride, chitosan, myricetin, sodium ascorbate, and galardin, have been used as adjunctive primers or incorporated into adhesive formulations. These agents have shown different degrees in improving dentin bonding through inter- and/or intra-fibril crosslinking [12,14,15].

Riboflavin (RF), or vitamin B2, is a photo-activated collagen crosslinking agent that exhibits high reactivity to produce free radicals or singlet oxygen (O_2_^−^ or ^1^O_2_) when irradiated with light in the spectral range from ultraviolet A (UVA) to blue light (BL) [16,17]. These free radicals break down some intrinsic weak cross-links in collagen fibrils, and the resulting unstable functional groups become active in forming new covalent crosslinks between adjacent collagen molecules [17]. Since its application in ophthalmology, RF combining with UVA treatment has demonstrated biocompatibility and efficacy in increasing corneal rigidity and resistance to biodegradation [18,19]. The introduction of RF/UVA in dentistry has also yielded promising results in both enhancing immediate resin–dentin bond strength and inhibiting MMPs [20]. In our previous studies [21,22], different RF/UVA protocols were evaluated regarding their efficacy in crosslinking dentinal collagen. Among them, the 0.1%RF/2-minUVA treatment was identified as the optimal protocol for improving resin–dentin bonding, possibly due to effective suspension of the collagen matrix in the HL. Although UVA has been proven effective as a photoactivation source for RF, safety concerns regarding its use and practicality should be considered. The cytotoxicity of high-intensity UV light needs to be carefully evaluated. Alternatively, Fawzy et al. suggested BL as another photoactivation source, which also significantly improves bond strength and resistance against collagenase [23]. Given that the wavelength of common clinical light-curing units ranges from 400 to 500 nm, they can also activate RF, converting it into an energized triplet excited state. In clinical dental offices, BL units are essential equipment for curing dental adhesive and restorative composites. Accordingly, BL could serve as an effective alternative to UVA due to its ready availability, ease of use, and safety.

The concentration of RF has also been identified as a major factor affecting the outcome of dentin bonding. According to Fawzy et al., 1% RF was superior to 0.1% RF under both UVA and BL in improving dentin bond strength and resistance to collagenase [23,24]. However, in our previous studies, 0.1% RF was found to be optimal for modulating the collagen matrix and facilitating resin infiltration [21,22]. Conversely, strong crosslinkers like 1% RF and glutaraldehyde rendered the collagen fibrils a barrier, thereby restraining the infiltration of resin monomers.

In this study, we designed a multifaceted evaluation to comprehensively investigate the effectiveness of RF combined with BL (RF/BL) in increasing collagen crosslinking and improving dentin bonding in four aspects:

(1) the effect on crosslinking collagen fibrils;
(2) the effect on enhancing mechanical properties of demineralized dentin;
(3) the ability to maintain collagen matrix suspension;
(4) the ability to inhibit dentinal MMPs.

Through these evaluations, we investigated whether RF/BL treatment could perform as effectively as RF/UVA treatment and determined its optimal operating conditions for improving dentin bonding.

## 2. Materials and Methods

In this study, six protocols were examined:

Control: no RF treatment;

RF0.1UV2: application of a 0.1% RF aqueous solution followed by 2 min of UV irradiation. This protocol was chosen for comparison due to its superior bond performances among various RF/UVA treatments in previous studies [20,21,22];

RF0.1BL1: application of a 0.1% RF aqueous solution followed by 1 min of BL irradiation;

RF0.1BL2: application of a 0.1% RF aqueous solution followed by 2 min of BL irradiation;

RF1BL1: application of a 1% RF aqueous solution followed by 1 min of BL irradiation;

RF1BL2: application of a 1% RF aqueous solution followed by 2 min of BL irradiation.

These protocols were evaluated for their effects on strengthening the collagen matrix, resisting enzymatic degradation, enhancing the integrity of the HL, and improving dentin bond strength. This study involved the collection of extracted human teeth as specimens, which was approved by the Institutional Review Board, National Cheng Kung University Hospital.

### 2.1. Absorbance Spectra of RF Material

A 0.01% aqueous RF solution was prepared by dissolving riboflavin 5′-phosphate sodium salt (Sigma-Aldrich, St. Louis, MO, USA) in deionized water. The absorption spectrum of RF was measured using a UV-Vis spectrophotometer (NanoDrop 2000, Thermo Fisher Scientific, Waltham, MA, USA).

### 2.2. Characterization of Light Sources

Two light irradiation units were used in this study. For UVA irradiation, a custom-made UVA unit (modified from Ultra-lite 1800E, Rolence, Taoyuan, Taiwan) was assembled. For BL irradiation, a dental quartz-tungsten-halogen light-curing unit (Optilux 501, Kerr, Bioggio, Switzerland) was used. The wavelength spectrum and irradiance of the two units were characterized with a laboratory-grade spectrometer (HR4000CG-UV-NIR, Ocean Optics, Dunedin, FL, USA) and a power meter (NOVA II Ophir, Tel Aviv, Israel), respectively.

### 2.3. Effect of RF/BL on Collagen Crosslinking

Sodium dodecyl sulfate–polyacrylamide gel electrophoresis (SDS-PAGE) was used to analyze collagen crosslinking. Type I collagen solution, isolated from bovine skin (3 mg/mL, Sigma-Aldrich), was neutralized by the dropwise addition of a buffer solution to reach a pH of 7.5. The final concentration of collagen solution was 2.25 mg/mL. A 5 µL aliquot of collagen solution was mixed with an equal volume of 0.1% or 1% RF solution and then subjected to BL irradiation for 1 or 2 min at a distance of 2 cm. A pure collagen solution mixed with deionized water was used as the control. All samples were mixed with 4x loading dye and heat-denatured at 100 °C. Each sample was then loaded onto an SDS-8% separating gel. After electrophoresis, the gels were stained with 0.1% Coomassie Blue R-250, processed in destaining buffer, and photographed. The densities of the γ, β, and α bands for each group in the electrophoretogram were quantified using the Gel Analysis function of ImageJ software (version 1.47, NIH, Bethesda, MD, USA).

### 2.4. Nanoindentation Test

A nanoindentation test was performed to evaluate the mechanical properties of demineralized dentin subjected to various RF treatments. Three extracted human molars were ground to expose the dentin. The coronal 2-mm portion of each tooth was sectioned and divided into six pieces for each group, then embedded in epoxy resin. The dentin surfaces were sequentially polished using 180- to 1500-grit silicon-carbide abrasive papers on a grinding machine (Ecomet 3; Buehler, Lake Bluff, IL, USA), followed by polishing with 1-μm diamond suspension. The flat surfaces were acid-etched for 15 s with 35% phosphoric acid (Ultraetch, South Jordan, UT, USA), and then subjected to the assigned surface treatments. The sample was stored in a dark environment before testing.

The nanoindentation test was performed with a computer-controlled nanoindenter (TI 700 ubi; Hysitron, Minneapolis, MN, USA) with a Berkovich diamond indenter. The indentation tests were carried out at 25 °C and a relative humidity of 45%. A loading test was performed by continuously loading and partially unloading for 30 cycles until the peak force reached 1000 μN. The elastic modulus (Em) and hardness (H) were determined from the load-displacement diagram.

### 2.5. Zymographic Analysis of MMP Inhibition

The method of zymographic analysis referred to the study by Cova et al. [20]. Ten freshly extracted human molars were obtained. Enamel, roots, and remnant pulp tissue were removed and washed with PBS supplemented with 1% penicillin-streptomycin. The dentin was powdered using a steel mortar and pestle at 4 °C and divided into six aliquots of 0.5 g. These powders were etched with 1% phosphoric acid for 15 min and rinsed with distilled water three times. The powders were then subjected to their assigned 0.5 mL RF solutions and light irradiations. Specimens were then resuspended in 0.5 mL extraction buffer at 4 °C for 24 h. The vials were centrifuged to collect the supernatants. Proteins in the supernatants were dialyzed through a 30-kDa membrane (Amicon ultra centrifugal filter, cat. no. UFC803024; Merck Millipore, Burlington, MA, USA), and stored at −20 °C until analyzed. Total protein concentration was determined using the Bradford assay. Proteins were electrophorized under non-reducing conditions on 8% SDS-polyacrylamide gels copolymerized with 1 g/L gelatin. Standard MMP-2 (from mouse, Sigma-Aldrich) was used as a positive control. After electrophoresis, the gels were washed with 2.5% Triton X-100 (Merck, Darmstadt, Germany) for 1 h and then incubated in developing buffer at 37 °C for 48 h. Finally, the gels were stained in 0.5% Coomassie Brilliant Blue R-250. Proteolytic activity was analyzed using ImageJ software.

### 2.6. Micro-Tensile Bond Strength (μTBS) Test

Sixty extracted human molars were collected and stored in 4 °C normal saline containing 0.02% sodium azide until use. These teeth were embedded in epoxy resin, ground flat to expose the dentin, and divided into six groups for the corresponding treatments. A standardized smear layer on the dentin surfaces was prepared by polishing with wet 180-, 320-, 400-, 600-grit abrasive papers serially. The dentin surfaces were etched with 35% phosphoric acid for 15 s, water-rinsed, and gently dried for 3 s to leave slightly moist. Subsequently, a single-bottle adhesive Singlebond (3M ESPE, St. Paul, MN, USA) was applied to the dentin surfaces by rubbing for 15 s and then light-cured for 10 s (Optilux 501, Kerr). 4mm-thick resin composite cubes (Z250, A2 shade; 3M ESPE) were built up in two layers, each light-cured for 20 s. After storage in 37 °C distilled water for 24 h, these teeth were sectioned perpendicular to the bonded surface to generate 1.0 mm × 1.0 mm resin–dentin beams with a low-speed saw under water cooling.

For each group, these microbeams were divided into two halves. One half received the μTBS test immediately after sectioning (early stage), while the other was subjected to enzymatic degradation. The latter was treated with a collagenase solution (Clostridiopeptidase A, Sigma-Aldrich) at 37 °C for 7 days, following the method described in our previous work [22]. The collagenase solution was changed every 24 h. For each group at two stages, twelve samples were included (*n* = 12 beams). The determination of sample size was based on our previous studies [21,22]. In those studies, a sample size of 12 per group was adequate to obtain a Type I error rate of 5% and a statistical power greater than 95%.

In the μTBS test, each beam was fixed with cyanoacrylate glue to a jig mounted on a universal testing machine (AG-1; Shimadzu, Kyoto, Japan). Individual resin–dentin beams were stressed under tension at a crosshead speed of 0.5 mm/min until failure. The μTBS was calculated by dividing the failure loads by the areas at the site of fracture. Statistical analysis was performed by a one-way ANOVA and a Duncan multiple comparison test at the significance level of *p* < 0.05. A student’s *t*-test was used to examine the statistical difference between two stages.

After the test, all fractured beams were dried in a desiccator for 1 day, mounted on stubs with conductive tape, and then transferred to a critical-point dryer for 30 min. Finally, all SEM specimens were sputter-coated with a thin gold film and examined by scanning electron microscopy (SEM) (JSM-6390 LV, JEOL, Tokyo, Japan) at 20 kV. The fracture pattern was classified as follows: A, 100% adhesive failure; Cd, 100% cohesive fracture in dentin; Cr, 100% cohesive fracture in composite resin. The area percentages of the three fracture patterns were calculated and statistically analyzed using a Chi-square test.

### 2.7. Micromorphology of the Hybrid Layer

In each group, three resin/dentin microbeams were used for examination of the resin/dentin interface morphology. The sectioned microbeams were stored in distilled water for 24 h. Subsequently, they were etched with 50% phosphoric acid for 5 s, rinsed with distilled water, then immersed in 10% sodium hypochlorite for 20 min to remove minerals and collagen from the dentin. These beams were fixed with 2.5% glutaraldehyde for 2 h and then dehydrated in ascending concentrations of ethanol from 20% to 100%. After the ethanol treatment, the specimens were dried in a critical-point dryer and sputter-coated with a gold–palladium alloy. Specimens were examined using SEM, and the resin/dentin interface was photographed. The adhesive layer thickness in each micrograph was measured and statistically analyzed by a one-way ANOVA and post hoc test.

## 3. Results

### 3.1. Absorbance of RF

**Figure 1:**
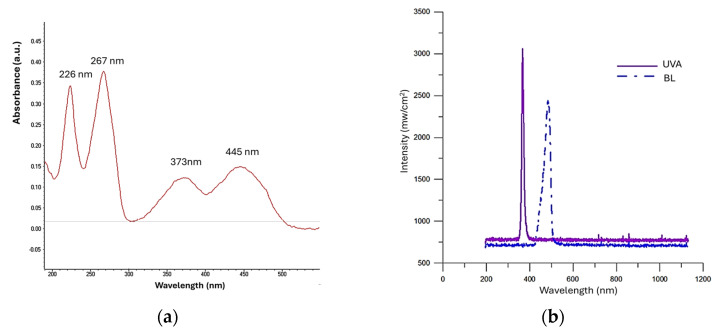
(**a**) The absorbance spectrum of riboflavin aqueous solution; (**b**) Spectra of UVA and BL units.

The absorbance spectrum of RF showed four peaks, characterized by three in the UV range (226, 267, 373 nm) and one in the BL range (445 nm) (Figure 1a).

### 3.2. Spectra of Light Sources

The wavelength range of UVA light was 360–400 nm, peaking at 375 nm (Figure 1b). The BL spectrum is wider (420–500 nm) and peaks at 450 nm. The irradiances of UVA and BL were 3320.5 ± 3.9 mW/cm^2^ and 2408.8 ± 2.5 mW/cm^2^, respectively.

### 3.3. Effect of RF/BL on Collagen Crosslinking

**Figure 2:**
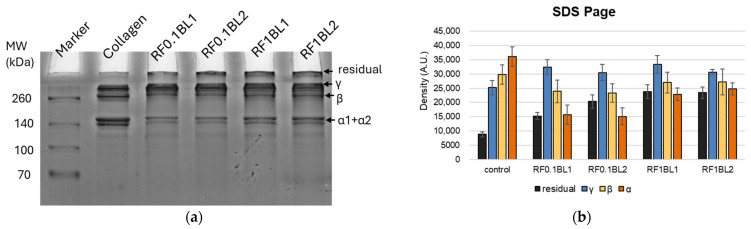
(**a**) Electrophoretogram (SDS-PAGE) of collagen receiving different treatments; (**b**) Density ratio plot of the SDS-PAGE. All the γ, β, and α bands are calibrated with the control (collagen).

The untreated collagen on the electrophoretogram showed the presence of the γ band (300 kDa), β band (260 kDa), and α1 and α2 bands (130 kDa) (Figure 2a). All RF/BL groups displayed residuals on the stacking gel, representing large crosslinked collagen molecules. The β bands in the RF0.1BL1 and RF0.1BL2 groups shifted slightly upward, indicating an increase in molecular weight. All RF/BL groups exhibited a reduced density of the α band (*p* < 0.005 for all groups), particularly in the two 0.1% RF groups. Light irradiation for 1 or 2 min did not result in any notable difference in these bands (Figure 2b).

### 3.4. Nanoindentation Test

**Figure 3:**
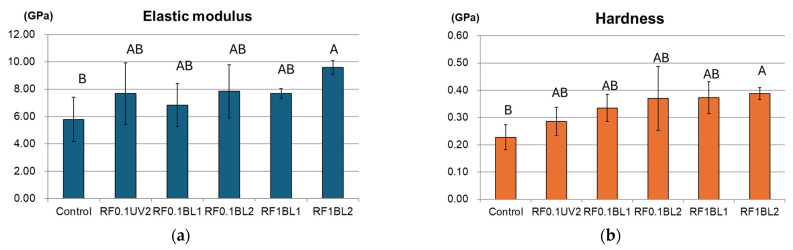
Results of the nanoindentation test. (**a**) Elastic modulus; (**b**) Hardness values. Identical letters represent no significant differences among treatments.

All RF/UVA and RF/BL groups showed higher Em and H values compared to the control (Figure 3). In the RF/BL groups, Em and H values increased with both RF concentration and irradiation time. Significant differences were observed only between RF1BL2 and the control (*p* = 0.027 for Em, *p* = 0.026 for H). No differences were found between RF0.1UV2 and any RF/BL group.

### 3.5. Zymography Analysis

**Figure 4:**
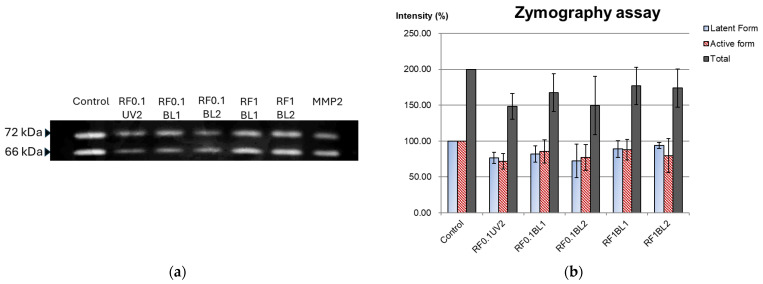
(**a**) Zymogram of the control and experimental groups; (**b**) The percentage of latent and active form of MMP2, and their total amounts.

In the zymography assay, proMMP-2 (latent form) and MMP-2 (active form) appeared at 72 kDa and 66 kDa, respectively (Figure 4a). Using the control as the standard (100%), all RF groups showed reduced MMP-2 activity (*p* < 0.05). No significant differences were observed within the RF groups (Figure 4b).

### 3.6. μTBS

For the initial test, RF0.1BL1 exhibited the highest μTBS, followed by RF0.1UV2 (Table 1). Other treatment groups, including RF0.1BL2, RF1BL1, and RF1BL2, showed μTBSs comparable to the control. Significant differences in the initial μTBS were observed between RF0.1UV2 and these groups (*p* < 0.05). After 7 days of enzymatic degradation, the bond strengths decreased in all groups. RF0.1BL1 still showed the highest μTBS but was not statistically different from RF0.1UV2 and RF0.1BL2. The μTBSs in these three groups were not significantly different from their initial values. RF1BL1 and RF1BL2 showed bond strengths comparable to the control, with both groups showing significant decreases in μTBSs (*p* < 0.05).

Regarding the fracture pattern, RF0.1UV2 and RF0.1BL1 exhibited the largest areas of cohesive fractures in the initial stage, significantly differing from the other four groups (*p* < 0.05) (Table 1). After enzymatic degradation, all groups exhibited an increase in adhesive fractures, with no cohesive dentin fracture observed. Among these, RF0.1UV2 showed the lowest proportion of adhesive fractures, while RF1BL2 exhibited the highest.

### 3.7. Morphology of Resin–Dentin Interfaces

**Figure 5:**
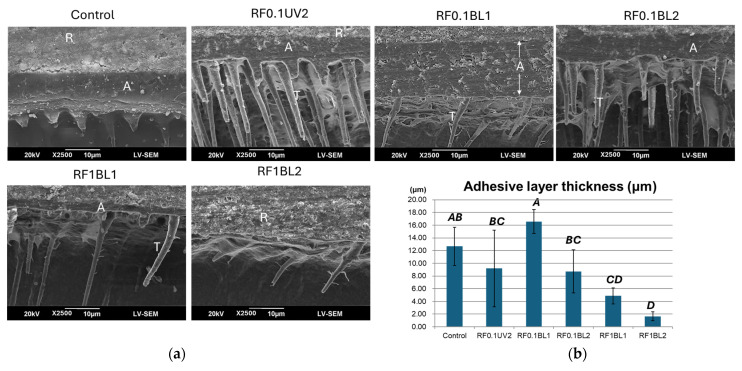
(**a**) Micromorphology of resin–dentin interfaces. (**b**) Average adhesive layer thicknesses (μm) in experimental groups. The same uppercase letters indicate no significant differences. Identical uppercase letters represent no significant differences among treatments. A: adhesive layer; R: resin composite; T: resin tags.

The integrity and morphology of the resin–dentin interface were evaluated using SEM micrographs (Figure 5). In the control group, a defective adhesive layer and short resin tags (less than 10 µm) were observed. The RF0.1BL1 group exhibited the thickest, though slightly defective, adhesive layer, along with some long resin tags. Both RF0.1UV2 and RF0.1BL2 groups displayed intact adhesive layers and long resin tags (more than 30 µm), with RF0.1BL2 even showing lateral branches on the resin tags. The RF1BL1 and RF1BL2 groups had very thin adhesive layers, with fewer resin tags compared to RF0.1UV2 and RF0.1BL2. In the RF1BL2 group, a separated interface between dentin and resin was observed.

The thicknesses of the adhesive layers were measured and compared (Figure 5). RF0.1BL1 had the greatest adhesive layer thickness, followed by the control, RF0.1UV2 and RF0.1BL2. The adhesive layers in the RF1BL1 and RF1BL2 groups were significantly thinner than in the RF0.1BL1 group (*p* < 0.001).

## 4. Discussion

The etch-and-rinse dentin adhesive systems are recognized as the gold standard in clinical restorative treatment, even after the evolution of successor systems, due to their high bond strengths. However, their performance may be impaired by insufficient resin monomer infiltration and the incompletely embedded collagen matrix. A certain rate of issues, including post-operative sensitivity, discoloration, marginal leakage, recurrent caries, and loss of the restoration, are associated with the adhesive strategy or methods employed [25]. Extrinsic crosslinking and reinforcement of collagen have been considered as tissue engineering approaches to improve the intrinsic properties of the substrate and ensure durable dentin bonding. Selective collagen crosslinkers are usually evaluated in several aspects: inducing the additional formation of inter- and intramolecular cross-links [26,27], increasing the mechanical properties of demineralized dentin [12,14], resisting biodegradation by MMPs and other collagenases [20,27,28], and ultimately improving dentin bond strength. Nevertheless, the role of crosslinking agents in enhancing dentin bonding also includes enhancing the quality of HL. Our previous study indicated that RF/UVA treatment functions in maintaining the collagen network in an expanded state, facilitating the inter-diffusion of resin monomers [21,22]. This study examined the effects of RF and BL irradiation on dentin bonding through a multifaceted approach. Since RF is an essential vitamin that plays major roles in metabolic processes, it poses no harm to human health. The use of blue light is also convenient and practicable. Except for certain cases involving deep cavities or root canals, contemporary BL devices are suitable for irradiating prepared dentin surfaces. Accordingly, this method could serve as a simple but effective adjunct treatment to address the deficiency of etch-and-rinse adhesives. The purpose was not only to identify the optimal protocol but also to elucidate the underlying action mechanism.

RF, combined with UVA or BL, has been approved as a promising treatment for strengthening the collagen network and the resulting HL, though the correct RF concentration and light irradiation conditions remain controversial. Fawzy et al. [23,24] have shown that RF/UVA led to a more significant improvement in the dentin bond strength compared to RF/BL, while 1% RF was superior to 0.1% RF. Other studies have also recommended UV-activated RF at 1–3% concentrations as effective and efficient treatments for dentin adhesion [29,30,31]. Conversely, pretreatment with UV-activated 0.1% RF or 0.1% RF-containing adhesive has been shown to improve resin–dentin hybridization by enhancing primer penetration [20,32,33,34]. In our work, 0.1% RF also showed the most significant effects in increasing dentin bond strength, reducing nanoleakage, and resisting enzymatic digestion among different treatments [21,22]. 1% RF showed less effective improvement, which is attributed to the strong crosslinking reaction that restrains the infiltration of resin monomers.

In this study, all the RF/BL groups showed collagen crosslinking effects, as evidenced by visible gelation changes, increased γ band intensity, and faded α bands in the SDS electrophoretogram. Compared to the RF1 groups, the RF0.1 groups showed greater reductions in α bands. This finding is possibly due to the distribution of RF in collagen solutions. During sample preparation, 1% RF caused aggregation upon contact with collagen solutions, which may impair its interaction with the remaining collagen molecules. Contrarily, 0.1% RF was more homogeneously distributed in the solution. The nanoindentation tests assessed the effect of collagen crosslinking on the stiffness of demineralized dentin. The result followed the rule of thumb that a higher concentration of RF and longer BL irradiation time induced higher stiffness of dentin. We could speculate that stiffness was affected by the degree of collagen crosslinking, and particularly influenced by BL irradiation time. RF0.1UV2 showed a similar elastic modulus as RF0.1BL2, but inferior hardness compared to all RF/BL groups. The results might be related to the slightly higher absorbance of RF at 445 nm (BL region) compared to 373 nm (UVA region).

Previous gelatin zymography and in situ zymography studies have reported that 0.1% RF combined with 1- to 5-min UVA exposure significantly reduced MMP-2 and MMP-9 activity compared to other crosslinkers [20,35]. However, contradictory studies have shown that 0.1% RF exhibited less resistance to collagenolytic challenges compared to 1% RF under UVA irradiation [23,24]. The zymogram in this study revealed the presence of proMMP-2 and MMP-2 but no MMP-9. The three 0.1% RF groups (RF0.1UV2, RF0.1BL1, and RF0.1BL2) were as effective as 1% RF in inactivating MMP-2. For the zymography analysis, this inhibitory effect was highly variable, depending on the protease examined and the method used, including the inhibitor titer [36]. We considered that the volume (500 µL) of 0.1% RF solution was sufficient to inactivate the endogenous protease released from the dentin powders, resulting in an effect comparable to that of 1% RF.

The results of the initial μTBS test indicated that RF0.1BL1 and RF0.1UV2 significantly improved μTBS, while the 1% RF groups showed values comparable to the control. After enzymatic degradation, the three 0.1% RF groups maintained higher μTBS values, with RF0.1BL1 exhibiting the highest value among all groups. In contrast, the μTBS values in the control and two 1% RF groups decreased significantly. The results corresponded to the micromorphological findings of the resin–dentin interfaces. In the SEM micrographs, RF0.1BL1 presented the thickest (>15 µm) adhesive layer and long resin tags. The adhesive layers of RF0.1UV2 and RF0.1BL2 groups were moderately thick, with abundant resin tags as well. These findings indicated successful resin–collagen hybridization, based on a well-suspended collagen matrix and sufficient infiltration of resin monomers, in these three groups. Conversely, the adhesive layer in the control group was porous. This was attributed to the collapse of the collagen matrix and the subsequent water perfusion impairing resin infiltration. RF1BL1 and RF1BL2 exhibited the thinnest adhesive layers and sparse resin tags, with some samples also showing interfacial debonding. Our previous study examined the orientation and morphology of collagen fibrils subjected to RF/UVA treatments using image analysis [21]. The results identified three types of collagen fiber alignments. Among them, 0.1% RF was found to be optimal for maintaining collagen fibrils in a projecting orientation after dentin demineralization. In contrast, the untreated control exhibited randomly aligned collagen fibers, while the strong crosslinker glutaraldehyde caused a tangled, enveloped collagen network. The thickest adhesive layers and abundant resin tags observed in RF0.1BL1 in the present study correspond to the expanded state of the collagen matrix, which facilitates resin monomer infiltration to form a robust resin–collagen network. On the other hand, 1% RF likely induced excessive collagen crosslinking, leading to fibril shrinkage and collapse, which impaired resin infiltration. These findings confirm that 0.1% RF is superior to 1% RF for dentin collagen treatment.

To summarize the findings of this study, BL-activated RF can crosslink dentinal collagen as effectively as UV in improving mechanical properties of demineralized dentin and inhibiting MMP-2. This method is clinically practicable, as BL devices are readily available in dental offices, and the treatment requires only 1 min. The effects of RF/BL treatments on improving dentin bonding are concentration-dependent. A 1% RF concentration significantly strengthened the dentin matrix, demonstrating its potency in stiffening collagen fibrils. However, three 0.1% RF treatments rendered milder crosslinking, optimally reinforcing the collagen, facilitating resin infiltration, and improving bond strengths both in the early stage and after biodegradation. The results suggest that combining 0.1% RF with 1-min BL irradiation enhances dentinal collagen crosslinking, strengthens the collagen matrix, and inhibits MMP activity. Ultimately, effective suspension of the collagen matrix appears to be the key to success for resin–dentin bonding.

## 5. Conclusions

This study examined the use of RF/BL to improve dentin bonding through a multifaceted approach. Based on the results, the following conclusions can be drawn:

1. All tested RF/BL protocols effectively crosslinked and strengthened the collagen fibrils, enhancing their resistance to collagenolytic challenges.
2. The application of 0.1% RF under BL significantly improved resin–dentin bond strengths and durability. In contrast, 1% RF may cause over-crosslinking, encapsulating the collagen matrix and impairing resin–dentin hybridization.
3. Combining 0.1% RF with 1-min BL irradiation could serve as an effective and clinically applicable treatment to enhance the bond quality of an etch-and-rinse dentin bonding system.

## Figures and Tables

**Table 1:** The μTBS values (MPa) and area percentages of different fractures in the experimental groups.

	Initial			Enz	
	μTBS	Fracture Pattern (%) (A/Cd/Cr)		μTBS	Fracture Pattern (%) (A/Cd/Cr)
Control	19.01 ± 6.18 ^Ba^	75/0/25		8.82 ± 7.95 ^Bb^	88/0/12
RF0.1UV2	26.80 ± 11.93 ^ABa^	42/12/46		21.96 ± 8.73 ^ABa^	81/0/19
RF0.1BL1	33.02 ± 11.14 ^Aa^	47/7/46		28.56 ± 11.64 ^Aa^	86/0/14
RF0.1BL2	23.13 ± 5.90 ^Ba^	77/0/23		18.42 ± 6.75 ^ABa^	93/0/7
RF1BL1	22.06 ± 4.41 ^Ba^	73/0/17		8.18 ± 7.93 ^Bb^	85/0/15
RF1BL2	21.30 ± 7.43 ^Ba^	88/0/12		8.54 ± 4.63 ^Bb^	97/0/3

Identical uppercase letters represent no significant differences among treatments. Identical lowercase letters represent no significant differences between initial and enzymatic degradation tests. A: adhesive failure; Cd: cohesive fracture in dentin; Cr: cohesive fracture in resin composite.

## Data Availability

The original contributions presented in the study are included in the article, further inquiries can be directed to the corresponding author.
